# The Influence of Dental Virtualization, Restoration Types, and Placement Angles on the Trueness and Contact Space in 3D-Printed Crowns: A Comprehensive Exploration

**DOI:** 10.3390/dj12010002

**Published:** 2023-12-19

**Authors:** Tsung-Yueh Lu, Wei-Chun Lin, Tzu-Hsuan Yang, Citra Dewi Sahrir, Yung-Kang Shen, Sheng-Wei Feng

**Affiliations:** 1 School of Dentistry, College of Oral Medicine, Taipei Medical University, Taipei 110, Taiwan; d204110005@tmu.edu.tw (T.-Y.L.); m249110002@tmu.edu.tw (C.D.S.); 2 School of Dental Technology, College of Oral Medicine, Taipei Medical University, Taipei 110, Taiwan; b210108004@tmu.edu.tw (T.-H.Y.); ykshen@tmu.edu.tw (Y.-K.S.); 3 Department of Dentistry, Wan-Fang Hospital, Taipei Medical University, Taipei 116, Taiwan; 4 Center for Tooth Bank and Dental Stem Cell Technology, Taipei Medical University, Taipei 110, Taiwan; 5 Division of Prosthodontics, Department of Dentistry, Taipei Medical University Hospital, Taipei 110, Taiwan

**Keywords:** dental virtualization, digital design, 3D printed, restoration types, placement angles, trueness

## Abstract

The current digital dentistry workflow has streamlined dental restoration production, but the effectiveness of digital virtual design and 3D printing for restorations still needs evaluation. This study explores the impact of model-free digital design and 3D-printing placement angles on restorations, including single crowns and long bridges produced with and without casts. The restorations are 3D printed using resin at placement angles of 0°, 60°, and 90°. Each group of samples was replicated ten times, resulting in a total of 120 restorations. The Root Mean Square Error (RMSE) value was used to evaluate the surface integrity of the restoration. In addition, the contact space, edge gap, and occlusal space of restorations produced by different processes were recorded. The results indicate that there was no significant difference in the RMSE value of the crown group (*p* > 0.05). Changing the bridge restoration angle from 0° to 90° resulted in RMSE values increasing by 2.02 times (without casts) and 2.39 times (with casts). Furthermore, the marginal gaps in the crown group were all less than 60 μm, indicating good adaptation. In contrast, the bridge group showed a significant increase in marginal gaps at higher placement angles (*p* > 0.05). Based on the findings, virtual fabrication without casts does not compromise the accuracy of dental restorations. When the position of the long bridge exceeds 60 degrees, the error will increase. Therefore, designs without casts and parallel placement result in higher accuracy for dental restorations.

## 1. Introduction

The digitalization process in dentistry has brought about many changes and advancements [1]. The next step is to discuss the technology of digital production and optimize the digital process. This comprehensive exploration encompasses topics such as fixed dentures, complete dentures [2], dental software [3], material mechanics [4], and related subjects. In the past, dental restorations were manually crafted by dental technicians [5]. The manufacturing process for dental restorations was complicated and time-consuming, requiring significant manpower. However, with advancements in medical and digital technology, dental practice has undergone a digital transformation. Digital changes have reduced many steps in traditional processes including impressions, plaster model making, wax-up engraving, and investment casting [6]. The digital process only needs to scan through the oral scanner to obtain the oral structure [5,7]. Restoration design is then carried out using dental computer-aided design/computer-aided manufacturing (CAD/CAM) software [8,9]. Finally, the dental restoration can be completed by transferring the digital restoration file to the milling machine or 3D-printing machine. Past studies have demonstrated that digitalization of the dental process improves production efficiency and enhances restoration accuracy [10]. However, it is essential to note that most of the digital production steps are operated by digital software, lacking physical contact from the oral scan to the completion of crown design. Consequently, digitalization still retains some uncertain factors compared to traditional manufacturing methods.

In clinical dental practice, some dental technicians will first scan the oral cavity files and make a solid resin model with a 3D printer. Finally, dental restoration is made using resin casts. However, errors may occur during file conversion when using 3D-printed dental resin casts. Therefore, the model will be at risk of deformation. Currently, the technology of dental digitization is quite stable [11]. Many people in dental clinics gradually cancel the process of the solid cast [12]. After obtaining the file of the patient’s oral scan, the dental restoration is designed and manufactured directly and installed in the mouth. However, the impact of the absence of casts in the fabrication procedures on the accuracy of restorations has not been reported. Additionally, the accuracy of the crown restoration design output is one of the key factors influencing the final result. Dental clinics often use 3D printers to produce models and restorations. Previous studies have shown that the placement angle of the print can affect the accuracy of single crown restorations [13]. Opting for a parallel orientation of objects to the platform is conducive to achieving dimensions of greater stability than those obtained with a vertical arrangement [14]. The printing of dental casts also demonstrates that the size of the dental arch and the bracket settings can cause changes in accuracy [15,16]. However, the effects on long-distance bridge restorations where multiple teeth are joined together remain unknown.

In the dental arch, crown restorations are limited to one side and do not extend across both the left and right sides [17]. A bridge restoration is the connection of multiple teeth that will span both sides of the dental arch. Therefore, the bridge forms a horseshoe shape [18]. Balancing the teeth position on both sides is crucial to create a lever effect, which can lead to deformation during the printing of the bridge restoration. Consequently, producing long-distance bridge restorations is currently a significant clinical challenge. The placement angle of 3D printing should be adjusted to suit different restoration types. Therefore, this study aimed to investigate the effect of model-free manufacturing and virtual design on the accuracy of dental restorations. By adjusting the placement angle of 3D printing, a more precise dental restoration can be achieved. The research will compare the trueness, contact space, and marginal gaps of single crowns and multiple bridges under different manufacturing conditions. The hypotheses of this study are as follows: (1) the without-casts fabrication process does not compromise the accuracy of dental restorations, and (2) different placement angles of 3D printing will influence the accuracy of the dental restorations.

## 2. Materials and Methods

### 2.1. Preparation of Dental Casts

In this study, dental standard models (Dental study model, Nissin Dental Product INC., Kyoto, Japan) were employed for fabricating crown and bridge restorations. Low speed (ultimate XL, NSK, Tokyo, Japan) was used to create 1.5 mm grooves on the buccal, lingual, mesial, distal, and occlusal surfaces of the tooth model. Then, these grooves were cut flatly to complete the preparation of the support ruler. Following clinical operation procedures, the first molar on the right side of the mandible was trimmed by an average of 1.5 mm to serve as the abutment tooth for the crown restoration. Similarly, all the upper jaw teeth of the model were uniformly trimmed to complete the abutment teeth for the long bridge restoration. Subsequently, a dental desktop scanner (Medit T510, Medit, Seoul, Republic of Korea) was utilized to create a digital file of the model, which was then used for restoration design.

### 2.2. Dental Prosthesis Design and 3D Printing

**Figure 1:**
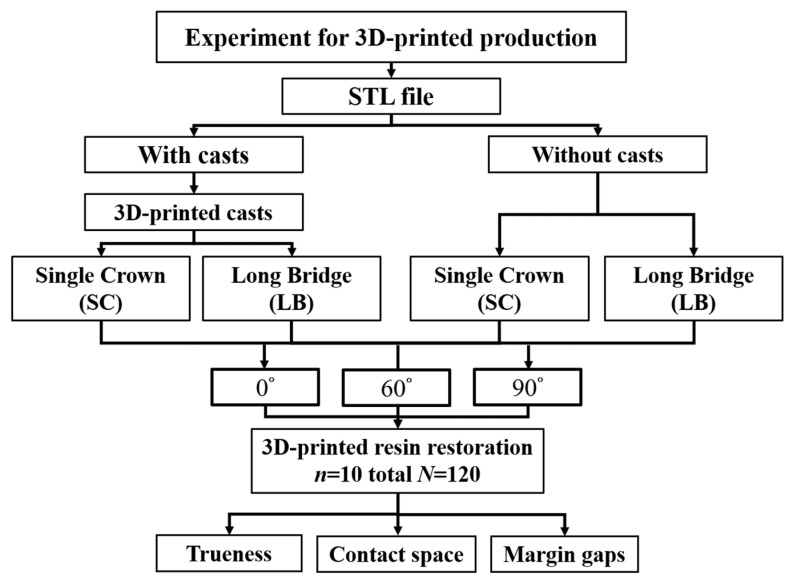
Schematic diagram of the study design and process.

This study primarily consists of two main groups: without casts and with casts (Figure 1). In the without-casts fabrication approach, restorations were directly designed from the digital data obtained through the dental desktop scanner (Medit T510, Medit, Seoul, Republic of Korea). On the other hand, in the with-casts group, we used digital files to incorporate brackets using software (Alpha 3D 3.0.3, Ackuretta, Taipei, Taiwan) and then printed the restorations using a 3D printer (Veribuild, Whip mix, Louisville, KY, USA). Dental model printing materials (Printin3D, Printin, Taoyuan, Taiwan) were used for printing at a resolution of 50 µm per layer. After printing, the model was immersed in 95% alcohol and cleaned using an ultrasonic oscillator (EASY Series, Elma, Singen, Germany) for 2 min. Following this, post-curing was conducted under UV light at a wavelength of 405 μm (Printin3D, Printin, Taoyuan Taiwan). Finally, a dental desktop scanner (Medit T510, Medit, Seoul, Republic of Korea) was utilized to scan and complete the digital files for the with-casts group.

**Figure 2:**
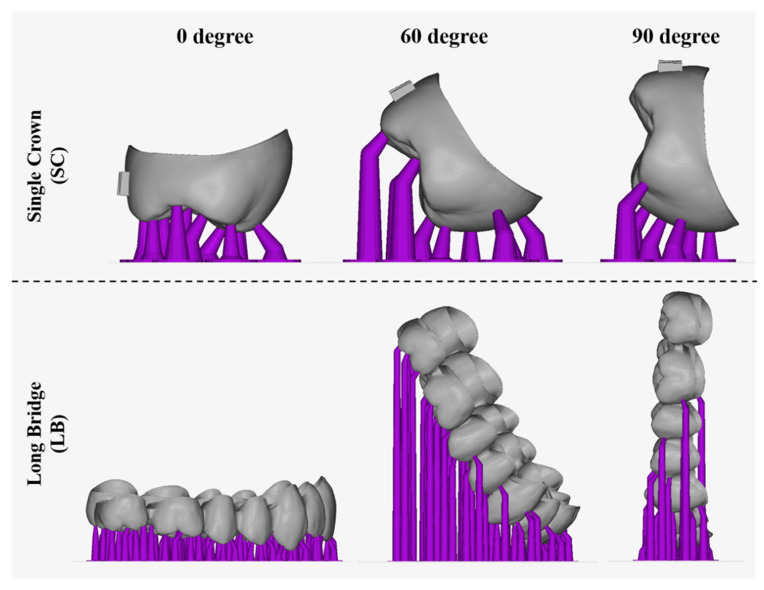
The schematic of the setup of a dental resin restoration from different angles.

Dental restorations were designed using inLab software (WS16, Dentsply Sirona, Charlotte, NC, USA). Two sets of restorations were created: one using digital files without casts and the other with casts made of resin. The design of crowns and bridges was carried out by the same dental technician, following standard clinical dentistry procedures. A total of four sets of design files for crown and bridge restorations were produced by the two manufacturing processes (Figure 1). In this study, the restoration files were placed on the design platform at different angles, and brackets were added (Figure 2). The 3D printing resin used for dental restorations (Printin3D, Printin, Taoyuan, Taiwan) consisted mainly of Urethane Acrylate (30–40%), Acrylic monomer (55–65%), 1.6-Hexanedinol Diacrylate (5–15%), and Photoinitiator (0–5%) [19]. Each group of samples was replicated 10 times, resulting in a total of 120 restorations. Finally, the restorations were cleaned and post-cured following clinical procedures to complete the restoration process.

### 2.3. Analysis of 3D Printing

3D printing limits the number of objects due to the size of the printing platform of the machine. Objects can increase the number of prints by changing the angle of placement. In this study, dental restorations were positioned on the platform at three different angles: 0°, 60°, and 90° (Figure 2). The maximum number of restorations was placed for evaluation using the automatic plate-setting function of the software (Alpha 3D 3.0.3, Ackuretta, Taipei, Taiwan).

### 2.4. Analysis of Trueness

This study primarily focuses on evaluating the impact of the virtual digital fabrication and 3D-printing placement angles on resin restorations. To achieve this, both crown and bridge restorations were scanned using a desktop scanner to generate digital files. These scanned files were then overlaid with the original design files using software (Medit Compare, Medit, Seoul, Republic of Korea) to visualize the differences in color distribution [20,21]. The mean Root Mean Square Error (RMSE) value of the matched profiles was computed as an estimate of trueness [13].

### 2.5. Analysis of Contact, Margin, and Occlusion 

**Figure 3:**
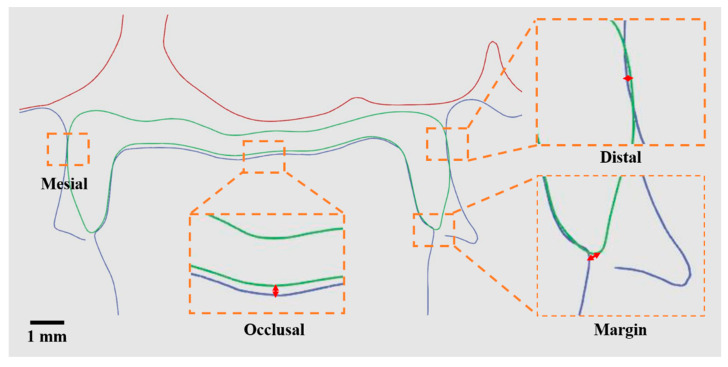
Schematic diagram of measuring the accuracy, contact, and margin of dental resin restorations by digital software. The orange line is an enlarged diagram. The red line is the distance.

In this study, the digital profile of the dental restorations was matched with the casts. The relationship between the restoration and the model was measured using digital software, which included assessing the contact space, marginal gaps, and occlusal space (Figure 3). To measure the contact space, the proximal and distal contact positions of the restoration were measured five times, and the average value was used for data comparison. Marginal gaps were mainly measured by evaluating the distance between the restoration and the edge of the abutment tooth. For this study, a total of 12 values were measured at three points on the proximal, distal, buccal, and lingual sides of the teeth for comparison. Additionally, the occlusal space was assessed by measuring the gap between the dental restoration and the abutment teeth at the occlusal surface. Five locations were measured for each group to obtain the average value. This study aimed to investigate the changes in dental bridge restorations in different arch positions. Therefore, the positions of five teeth, including the middle incisors (#11 and #21), first premolars (#14 and #24), and second molars (#17 and #27), on the left and right sides of the maxillary arch were measured for analysis.

### 2.6. Statistical Analysis

The data are presented as the mean ± standard deviation (SD) from 10 replicate samples. Statistical analysis was performed using JMP 16 software (Statistics Analysis System, Cary, NC, USA). The normal distribution of the data was assessed using the Shapiro–Wilk test. Two-way ANOVA was used to identify the variables (trueness, contact, margin, and occlusion). To assess significance, Tukey’s Honestly Significant Difference (HSD) post hoc test was conducted, with *p* < 0.05 considered statistically significant.

## 3. Results

### 3.1. Analysis of 3D Printing

Table 1 provides information on the placement angles and maximum bracket numbers for different dental restorations. Specifically, there were 45 crown restorations placed at 0°, 45 crown restorations placed at 60°, and 48 crown restorations placed at 90°. For bridge restorations, there were three groups placed at 0°, four groups placed at 60°, and seven groups placed at 90°. It is worth noting that all groups achieved the maximum number of placements at 90° (Table 1).

### 3.2. Analysis of Trueness

**Figure 4:**
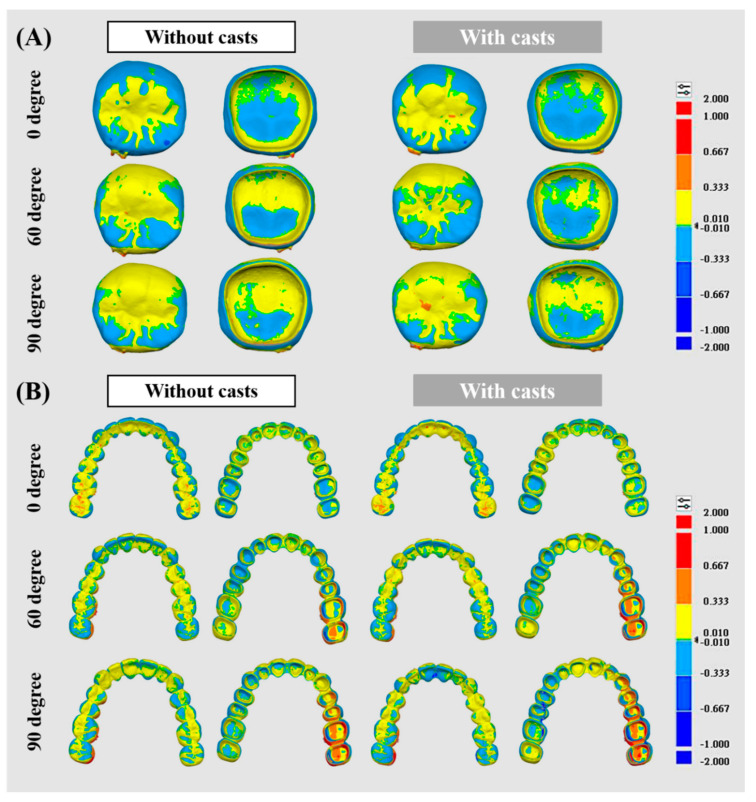
Surface 3D trueness color distribution for different resin restorations compared to each other. (**A**) Single crown. (**B**) Long bridge.

**Figure 5:**
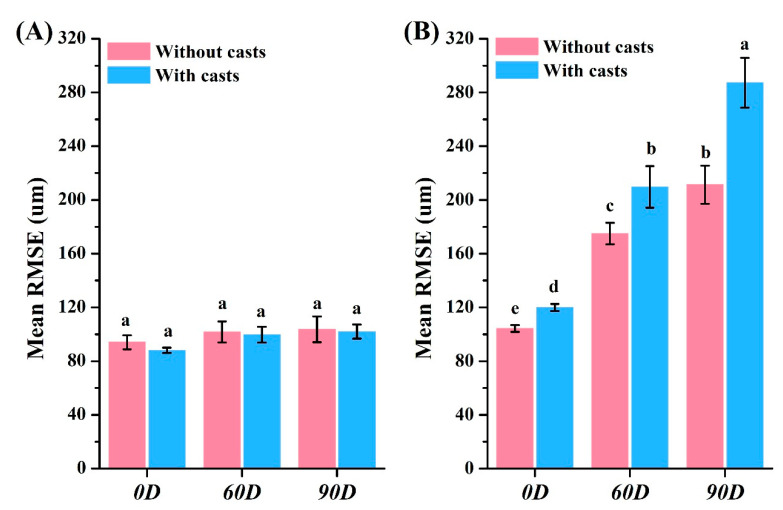
Accuracy analysis of different resin restorations. The RMSE value of the (**A**) crown and (**B**) bridge. Means with different letters were significantly different (*p* < 0.05, mean ± SD, *n* = 10).

The crown groups displayed similar surface realism with and without models, showing a color shift from blue at 0 degrees to yellow at 90 degrees (Figure 4A). However, significant differences were observed between the without-casts and with-casts groups for bridge restorations, with the latter exhibiting a biased red color distribution (Figure 4B). The RMSE results aligned with these trends. For crown restorations, the RMSE values showed no significant differences (*p* > 0.05) across all groups (88.0 μm to 103.7 μm) (Figure 5A). In contrast, bridge restorations without casts showed significant differences in the RMSE at different angles (104.3 μm at 0 degrees, 175.0 μm at 60 degrees, and 211.3 μm at 90 degrees, *p* < 0.05) (Figure 5B). The group with casts also demonstrated significant differences (*p* < 0.05) in the RMSE at different angles (120.0 μm at 0 degrees, 209.7 μm at 60 degrees, and 287.3 μm at 90 degrees). The RMSE of bridge restorations with casts increased by 13.0% at 0 degrees, 19.8% at 60 degrees, and 35.9% at 90 degrees, respectively. In the without-casts group, bridge restorations showed an increase of 67.7% at 60 degrees and 102.5% at 90 degrees compared to 0 degrees. Similarly, the with-casts group exhibited an increase of 74.7% at 60 degrees and 139.4% at 90 degrees compared to 0 degrees, respectively.

### 3.3. Analysis of Contact, Margin, and Occlusion

**Figure 6:**
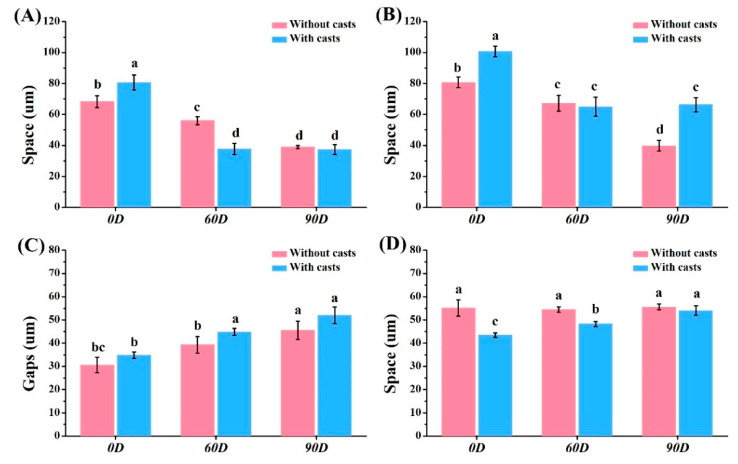
Contact space and margin space analysis of single crown. (**A**) Mesial contact space. (**B**) Distal contact space. (**C**) Marginal gaps. (**D**) Occlusal space. Means with different letters were significantly different (*p* < 0.05, mean ± SD, *n* = 10).

**Figure 7:**
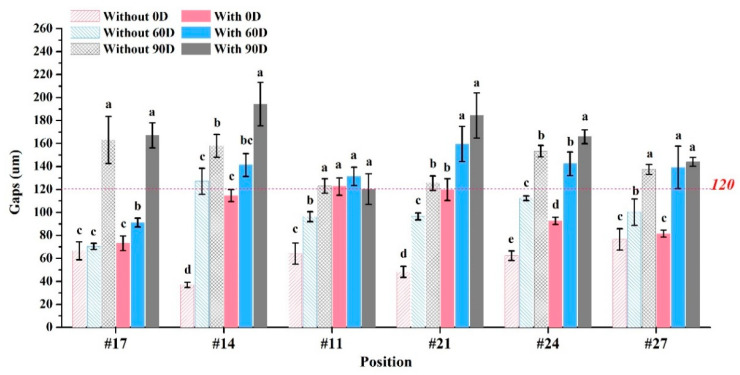
Marginal gaps analysis of long bridge. Means with different letters were significantly different (*p* < 0.05, mean ± SD, *n* = 10).

**Figure 8:**
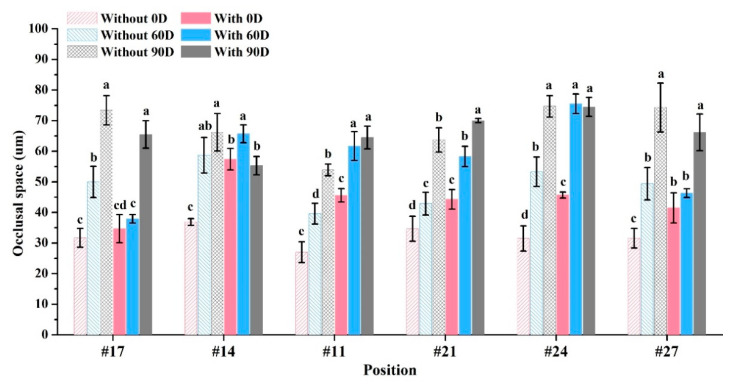
Occlusal space analysis of long bridge. Means with different letters were significantly different (*p* < 0.05, mean ± SD, *n* = 10).

Mesial contact space for crown restorations: 39.0–68.3 μm (without casts) and 37.3–80.7 μm (with casts) (Figure 6A). Distal contact space: 39.7–80.7 μm (without casts) and 65.0–100.7 μm (with casts) (Figure 6B). Marginal gaps: 30.6–45.5 μm (without casts) and 34.8–52.0 μm (with casts) (Figure 6C). Occlusal space: 54.5–55.6 μm (without casts) and 43.5–54.0 μm (with casts) (Figure 6D). Marginal gaps for bridge restorations without casts: 37.0–163.1 μm (Figure 7) and with casts: 73.1–194.2 μm (Figure 7), both showing significant differences (*p* < 0.05). Occlusal space for bridge restorations without casts: 27.0–74.7 μm (Figure 8) and with casts: 34.7–74.5 μm (Figure 8), both exhibiting significant differences (*p* < 0.05).

## 4. Discussion

Caries are a common dental condition in clinical dentistry, leading to permanent damage to the tooth surface [22,23,24]. Clinical dentistry must employ customized dental restorations to restore the eroded parts of teeth, ensuring the restoration of both their beauty and functionality [21]. In the past, traditional dental restorations were made using solid models. However, current advances in dental scanners, dental restorative software, dental CAD/CAM systems, and 3D printers have significantly enhanced the advancement of dental medical [25,26]. The primary goal of this digitization is to increase the precision and efficiency. Dental restorations are mainly divided into two stages: design and manufacture. Regarding the design stage, digital software and artificial intelligence can greatly save time [27]. Whether using a CAD/CAM system (subtractive processing) or a 3D printer (additive processing), relative production time must be sacrificed in order to improve accuracy [28]. A particular breakthrough in improving performance would be how 3D-printing technology can place more objects on the same plane. In this study, different crown restorations were placed on the 3D-printing platform at various angles to evaluate the maximum capacity (Table 1). Since the length and width of the single crown restorations are similar, changes in the angle did not significantly affect the quantity. However, changes in the placement angle had a significant impact on the number of bridges. When the placement angle was changed from 0 degrees to 90 degrees, the number of bridges increased by 133.3%. This is because increasing the distance across the arch significantly increases the area of the bridge. When the bridge is perpendicular to the plane (90 degrees), the contact area is reduced, thereby increasing the capacity (Figure 2).

The manufacturing process of dental restorations varies from type to type [29]. This study aimed to explore the impact of virtual design without casts and 3D-printing angles on the accuracy of different restoration types. The differences in restorations under various conditions can be observed through the distribution of surface color. A red color indicates a larger restoration, while a blue color indicates a smaller one (Figure 4). The crown restorations showed no significant changes under different conditions (Figure 4A). It is interesting to note that there was a slightly larger crown observed when there was a more yellow distribution on the surface at a 90° angle. On the other hand, the color distribution of bridge restorations was significantly affected by the angle (Figure 4B). The Root Mean Square Error (RMSE) values were calculated by the software to assess the surface realism aligned with the color distribution result (Figure 5). The RMSE values for the crown restorations were not significantly different (*p* > 0.05). However, there was a significant difference (*p* < 0.05) in the RMSE values of the bridge restorations under different conditions. The bridges showed fewer errors in the without-casts condition. Moreover, the angle of placement significantly affected the accuracy. When the bridge restoration angle changed from 0° to 90°, the RMSE values increased by 2.02 times (without casts) and 2.39 times (with casts). This might be due to the front teeth near the platform solidifying and forming first during the printing process, while the printing of the rear teeth on both sides had not been completed, resulting in errors. Notably, the most obvious difference in the color distribution was observed in the rearmost teeth on both sides of the bridge (#17 and #27). Previous studies have shown that a measurement error of <200 μm in RMSE values is clinically acceptable [30]. Therefore, even though a large number of prints can be obtained, bridge restorations placed at 90 degrees are still clinically unacceptable. The choice of a 3D printer and the characteristics of the resin material may offer a potential solution to address the constraints associated with 90-degree printing. By employing faster printing speeds and more stable resin formulations, it becomes conceivable to mitigate the challenges associated with deformation arising from prolonged printing durations. Certainly, further validation is required to elucidate the optimal strategy for large-scale production. In addition, Lu et al. showed that parallel 3D printing has better zirconia strength than vertical printing [31]. It is demonstrated that parallel contact between the restoration and the placement platform is more suitable for 3D-printing designs.

The teeth are closely spaced above the alveolar bone with adjacent teeth in the oral cavity [32]. They are responsible for grinding and chewing food between the upper and lower jaws [33]. If there is excessive contact between the teeth, the restoration will not fit properly on the abutment teeth in the mouth. On the other hand, too little contact may lead to food blockage. The influence of crown restorations with and without the model on the contact relationship was found to be less significant than that of the placement angle (Figure 6A,B). Although there were significant differences (*p* < 0.05) among all samples, they all exhibited good contact relationships. Furthermore, it was observed that the larger the printing angle, the smaller the gap between the crown and adjacent teeth. This implies that increasing the printing angle results in a larger crown size.

The crown restoration requires a careful evaluation of its contact relationship with adjacent teeth. Additionally, the margin clearance between the restoration and the abutment tooth needs to be considered. The literature indicates that oral bacteria often invade the crown margins causing secondary caries [34,35]. Therefore, the marginal gap of dental restorations must be less than 120 μm to meet clinical requirements [36]. In this study, crown restorations exhibited process marginal gaps that were smaller in the without-casts group compared to the with-casts group (*p* < 0.05). However, the marginal gaps of crown restorations in each group were all less than 60 μm, demonstrating a good fit (Figure 6C). For long bridges, the marginal gap results significantly differed from crowns (Figure 7). The printing angle notably influenced the marginal gaps of the bridge. The deformation of the bridge surface prevented proper loading on the casts, resulting in an increase in the gap. When printing a bridge restoration spanning the left and right sides, the deformed parts were mainly located at both sides of the rear end. Placing it at a vertical 90-degree angle caused severe errors, making it unusable. These results align with the literature suggesting that bridges must be parallel to the printing plane to maintain proper contact and reduce errors [16].

The purpose of this study was to determine whether the increased space was due to poor fitting of the restoration. Therefore, the occlusal space between the restoration and the abutment teeth was measured (Figure 6D and Figure 8). The results for both the crown and bridge restorations showed a space smaller than the original 90 μm. Crown restorations exhibited a lower occlusal space compared to bridges. For bridge restorations, the with-casts group had a higher occlusal space than the without-casts group at different placement angles (*p* < 0.05). This was because the with-casts group experienced significant deformation and could not be correctly loaded on the abutment teeth. As a result, the marginal gaps of the bridge restoration in the with-casts group were not deformed due to an increase in the transverse dimension. The deformation occurred when the entire restoration could not be loaded correctly onto the abutment teeth, leading to premature contact and increased space. Thus, the without-casts digital restoration does not compromise accuracy. Although designing dental restorations placed at 90 degrees can increase the number of prints, it significantly reduces the accuracy. The above results support the study’s hypothesis that (1) the without-casts fabrication process does not reduce the accuracy of dental restorations and (2) different placement angles of 3D printing will affect the accuracy of dental restorations.

## 5. Conclusions

The outcomes derived solely from this study suggest that opting for virtual fabrication without casts does not compromise the accuracy of dental restorations. Specifically, the angle of 3D-printing placement exhibited no impact on the accuracy of single crowns. However, it is crucial to note that substantial errors were identified when placing bridge restorations at angles exceeding 60°. Consequently, for attaining a heightened level of precision in dental restoration, it is advisable to employ a virtual design coupled with a placement angle of 0 degrees.

## Figures and Tables

**Table 1:** Number of restorations with different placement angles.

Angle (Degree)	Single Crown	Long Bridge
0	45	3
60	45	4
90	48	7

## Data Availability

Data are contained within the article.
